# Greater Power but Not Strength Gains Using Flywheel Versus Equivolumed Traditional Strength Training in Junior Basketball Players

**DOI:** 10.3390/ijerph18031181

**Published:** 2021-01-29

**Authors:** Marko D. M. Stojanović, Mladen Mikić, Patrik Drid, Julio Calleja-González, Nebojša Maksimović, Bogdan Belegišanin, Veselin Sekulović

**Affiliations:** 1 Advanced Rehab & Conditioning Lab, Faculty of Sport and Physical Education, University of Novi Sad, 21000 Novi Sad, Serbia; mmmikac@gmail.com (M.M.); patrikdrid@gmail.com (P.D.); nebojsam@uns.ac.rs (N.M.); belegisaninb@gmail.com (B.B.); veselinsekulovic@gmail.com (V.S.); 2 Faculty of Education and Sport, University of the Basque Country (UPV/EHU), 01007 Vitoria-Gasteiz, Spain; julio.calleja.gonzalez@gmail.com

**Keywords:** isoinertial training, strength training, vertical jump, change of direction ability

## Abstract

The main aim of the present study was to compare the effects of flywheel strength training and traditional strength training on fitness attributes. Thirty-six well trained junior basketball players (n = 36; 17.58 ± 0.50 years) were recruited and randomly allocated into: Flywheel group (FST; n = 12), traditional strength training group (TST; n = 12) and control group (CON; n = 12). All groups attended 5 basketball practices and one official match a week during the study period. Experimental groups additionally participated in the eight-week, 1–2 d/w equivolume intervention conducted using a flywheel device (inertia = 0.075 kg·m^−2^) for FST or free weights (80%1 RM) for TST. Pre-to post changes in lower limb isometric strength (ISOMET), 5 and 20 m sprint time (SPR5m and SPR20m), countermovement jump height (CMJ) and change of direction ability (*t*-test) were assessed with analyses of variance (3 × 2 ANOVA). Significant group-by-time interaction was found for ISOMET (F = 6.40; *p* = 0.000), CMJ (F = 7.45; *p* = 0.001), SPR5m (F = 7.45; *p* = 0.010) and T test (F = 10.46; *p* = 0.000). The results showed a significantly higher improvement in CMJ (*p* = 0.006; 11.7% vs. 6.8%), SPR5m (*p* = 0.001; 10.3% vs. 5.9%) and *t*-test (*p* = 0.045; 2.4% vs. 1.5%) for FST compared to the TST group. Simultaneously, th FST group had higher improvement in ISOMET (*p* = 0.014; 18.7% vs. 2.9%), CMJ (*p* = 0.000; 11.7% vs. 0.3%), SPR5m (*p* = 0.000; 10.3% vs. 3.4%) and *t*-test (*p* = 0.000; 2.4% vs. 0.6%) compared to the CON group. Players from the TST group showed better results in CMJ (*p* = 0.006; 6.8% vs. 0.3%) and *t*-test (*p* = 0.018; 1.5% vs. 0.6%) compared to players from the CON group. No significant group-by-time interaction was found for sprint 20 m (F = 2.52; *p* = 0.088). Eight weeks of flywheel training (1–2 sessions per week) performed at maximum concentric intensity induces superior improvements in CMJ, 5 m sprint time and change of direction ability than equivolumed traditional weight training in well trained junior basketball players. Accordingly, coaches and trainers could be advised to use flywheel training for developing power related performance attributes in young basketball players.

## 1. Introduction

It has been acknowledged in the scientific literature that strength training produces several morphological and neural adaptive changes in the human body, including increases in muscle’s cross-sectional area, muscle fiber pennation angles and musculotendinous stiffness as well as motor unit recruitment, rate coding (firing frequency), synchronous motor unit activity and neuromuscular inhibition [1]. These types of adaptations enable an increase in strength and power—both of them have been extensively proven to be related to sport performance across a continuum of sports events [2]. Consequently, strength training has become a cornerstone of strength and conditioning programs for athletes [3]. In addition, optimizing the load and time spent in strength training may be one of the most important considerations for strength and conditioning coaches (especially in team sports), where success is multifaceted and with a broad spectrum of physical, physiological, technical and tactical abilities that need to be targeted regularly in the training process and integrated periodization [4]. Consequently, in both sport science and everyday practice there is a need for elucidating and incorporating effective but also time sparing strength training methods [5]. In this vein, many different strength training methods have been presented in the past, including the use of free weights, kettlebells, elastic bands and resistance training machines [6]. These different traditional strength training methods, including both eccentric and concentric muscle actions, are prescribed based on concentric force parameters with propensity to underload the lengthening phase of movement as muscle produces more force during eccentric phase of movement [7]. There is a growing body of research asserting that strength training programs which adequately load the lengthening phase of movement, called eccentric training, might induce superior neuromuscular adaptations (faster cortical activity, inversed motoneuron activity pattern, improved muscle-tendon unit morphology and structure) compared with traditional strength training. In addition, there is increasing evidence in recent scientific literature implying that eccentric strength training is a potent stimulus for boosting physical performance [8,9], with flywheel iso-inertial resistance training especially highlighted recently for its efficiency in both performance and clinical settings [10] as well as specificity [11].

Concisely, flywheel training is a relatively new training method consisting of participants accelerating a flywheel during concentric phase of movement with kinetic energy returned during the eccentric phase of movement, thus requiring significant eccentric muscle action (eccentric overload) to slow the flywheel. This presents an alternative means of providing external load in resistance exercises which can be achieved by flywheel resistance [12]. Flywheel training enables overload in the eccentric phase, by resisting the eccentric force later in the eccentric range of motion [13]. Considering performance outcomes in the athletic population, eight to eleven weeks of flywheel training with one/two sessions a week has been found effective to enhance countermovement jump height (CMJ), change of direction ability and linear sprint in young and adult soccer players [14,15,16,17,18]. Furthermore, literature found six and seven weeks of flywheel training (two-three and one session per week, respectively) to be a robust tool to significantly enhance CMJ, squat jump, 20 m sprint, change of direction (*t*-test) and maximal strength [19] as well as maximal strength (Half squat 1 RM) and 20 m sprint [20] in professional handball players. Interestingly, the effects of flywheel training on performance outcomes in basketball are scarce. To the best of the authors knowledge, only one study [21] reported significant improvements in countermovement jump and squat power after implementing one session a week of flywheel training (four sets of eight repetitions of the squat, 24 weeks) in a sample of 26 regional level adult basketball players (males and females). Change of direction, muscular strength, vertical jumping ability and repetitive short-distance sprints are all important fitness attributes required for the physical demands of a basketball game [22]. In addition, the relevance of performing explosive and fast movements, such as sprints, jumps and change of direction has increased in modern basketball [22]. Finally, lower body strength has been extensively reported to be related to lower body power performance [23]. Therefore, it is of interest for both sport scientists and basketball practitioners to elucidate the effects of innovative training methods for power and strength development in basketball.

Although strength and conditioning coaches use various methods to develop neuromuscular factors in youth basketball players [24], no studies to date, as far as we know, have investigated the effects of flywheel training on strength and power attributes in young basketball players. It should be recognized that the continuation of habitual team sport practice during puberty was proven to induce substantial improvements in lower body strength per se, without additional resistance training performed [25]. Consequently, the inclusion of the control group with regular basketball practice would improve clarity of whether performance adaptations are consequence of strength training or specific sport training linked to the possible growth and muscular development. This was not the case with previous similar investigations conducted on young soccer players [14,15]. Furthermore, to the best of our knowledge, there are no studies with relatively old (U-18), highly trained and resistance training-experienced adolescents that have compared the effects of continuing with specific sport practice or including flywheel or traditional strength training to regular basketball practice. Recently, meta-analysis exploring the flywheel training performance effects revealed that most interventions caried out on 5 to 10 weeks training period [13]. Further, in vertical inertial flywheel training, similar to our research design [14,19,26], differences in strength and power performance in 6- and 8-week training period were found. As a result of this analysis, an eight-week training period is consistent with previous research.

Taking all aforementioned, the main aim of this study was to compare the in-season effects of eight week of equivolumed flywheel vs. traditional strength training on lower body strength, countermovement jump, *t*-test and 5 and 20 m sprint performance in well-trained young basketball players. We hypothesized that flywheel training will produce superior effect in all observed fitness attributes.

## 2. Materials and Methods

### 2.1. Participants

Thirty-six well trained junior male basketball players volunteered to participate in the study and were randomly assigned to 3 groups: the first experimental group (FST; n = 12; age = 17.58 ± 0.52 years; height = 190.54 ± 4.98 cm; body mass = 75.53 ± 5.43 kg; training experience = 6.17 ± 1.19 years) which performed strength training on a flywheel training device ((D11 full, Desmotec, Biella, Italy), the second experimental group (TST; n = 12; age = 17.52 ± 0.58 years; height = 190.58 ± 6.56 cm, body mass = 78.78 ± 8.01 kg; training experience = 6.92 ± 2.88 years) which performed traditional free weights strength training and the control group (CON; n = 12; age = 17.56 ± 0.54 years; body mass = 192.81 ± 3.99 cm; weight = 80.00 ± 8.76 kg; training experience = 6.58 ± 1.38 years) which maintained regular basketball practice.

All players where regional level, from Novi Sad (Serbia) and played for the teams contesting in the junior league of Vojvodina province during the season in which the investigation took place. All players had basketball training experience of a minimum of 4 years, without lower limb injury or illness 4 months prior to the study. During the program, all participants had 5 basketball trainings (90 min per training) and one game a week. In addition, participants were all familiar with resistance training regularly exploited throughout the season, but without previous experience with flywheel device. The requirements and obligations during the study were explained to all participants, as well as the purpose of the research. Each participant could withdraw from the research at any time. No players reported injuries throughout the study duration and no one withdraw from the research. The study fits the Declaration of Helsinki (2008), actualization in Fortaleza 2013 [27], for medical research involving human participants.

The study protocol was reviewed and approved by the ethics committee of the University of Novi Sad, Serbia. (Ref. No. 44-01-02/2019-3). All participants voluntarily accepted to enroll in the study and signed an informed consent, while parents or legal representatives signed for underage subjects.

### 2.2. Study Design

The experimental program was organized during the second part of the competition period, in March and April 2019. Initial testing was organized seven days prior to the first practice session, and after testing players from the FST and TST groups attempt two sets with six–eight repetitions on an isoinertial device (FST group) and with weights (TST group) in order to familiarize with the training protocol. Three days before starting the program, both experimental groups had a second familiarization training with exercises on an isoinertial device and with free weights. Supervised strength training for the experimental groups was conducted during the morning hours in the lab facility at a Faculty of sport and physical education, University of Novi Sad, supplied with all necessary equipment (flywheel, bars, plates, elastic bands…). All participants were supervised by PhD students with extensive strength training experience to help ensure high quality training sessions. The sessions were performed on every Tuesday, Wednesday and Thursday, in groups of no more than 6 players and monitored by at least two PhD students at all times. Three to six days after the intervention period final testing was conducted, identical with initial one considering time of testing, order and protocols of testing procedures and examiners. All participants were strongly advised to avoid any strenuous activity 24 h before testing. The control group did not receive any additional training apart from regular basketball trainings and weekend-games during the intervention. During the week, but not on the same day as the experimental program, one basketball training session, was supplemented with bodyweight strength training for all groups. This training was regularly implemented throughout the season, at the beginning of the training, lasting 25 to 30 min. The participants were not allowed to take stimulants, or any other substances for improving performance during the study.

### 2.3. Measurements

Anthropometric measurements were taken by an International Society for Advancement in Kinanthropometry (ISAK) level three anthropometrist, following the standard procedures prior to initial testing [28]. The height and body mass technical error of measurement (TEM) was less than 0.02%, and were measured with an SECA (Seca GmbH, Hambrug, Germany) measuring rod, (precision of 1 mm; range: 130–210 cm) and an SECA model scale (precision of 0.1 kg; range: 2–130 kg).

Prior to initial testing, data on training experience and anthropometric measures of standing height and body weight were taken for each subject. The lower extremity isometric strength test (ISOMET) was performed with peak force measured on an isoinertial device (D11 full, Desmotec, Biella, Italy). The participant was connected to the device by a strap with one end tied to the device and the other to a waistcoat worn by the participant. The strap was tightened not to allow the respondent to move up. The Desmotec device has two contact panels that are connected to a computer equipped with the software (D.Soft, Desmotec, Biella, Italy). The participant stands in a semi-squat position, flexion at 100 degrees angle, and his hands are placed on his hips. At the sign, the subject exerts pressure on the plates for 10 s, maximum voluntary isometric contraction. The contact panels measure the force that the participant produces and which is read on the computer. The test was done twice, with a rest period of 2 min, and the better result, expressed in kilograms, was recorded. Good test-retest reliability (α = 0.889) was found for this parameter. 

Countermovement jump test—CMJ—was conducted according to Bosco protocol [29] on a contact platform Just Jump, Probotics, USA. During the CMJ, all participants were instructed to start with upright posture and their hands on their hips. After swift downward phase to semi squat position, participants jump up in the air maximally keeping hands on their hips and landing in an upright position with their knees extended. Three attempts were allowed, with 45 s of passive recovery between trials. The best jump performance was registered and used for further analysis. CMJ is characterized by a very low variability between tests (coefficient of variation of 3.0%) [30], with excellent test-retest reliability (α = 0.918) found in our study. 

Subjects performed a 20 m sprint test, with 5 m split time and times were recorded using light gates (Microgate—Witty, Italy). Two submaximal efforts were included at the end of specific warm up, followed two 20 m sprint trials, with two minutes of passive recovery between trials. After a specific warm-up, including the 2 submaximal efforts (around 90% of max speed), two trials were completed. The subject started from the crouched position with the front foot positioned 0.3 m behind the first timing gate, where players started voluntarily and accelerate maximally to the finish line. During the test, the participants were verbally encouraged to run with maximum effort. The better results were used for further statistical analysis (SPR5 m and SPR20 m). The 20 m sprint test has demonstrated high level of reliability in our study (α = 0.901 and α = 0.914 for 5 m and 20 m sprint, respectively), which is similar to previous study findings [31].

Agility *t*-test was conducted according to Semenick [32]. The participants starts with front foot positioned 0.3 m before the light gate. The test includes forward running, shuffling sideways and in the end backwards running. The trial was not counted if the player crossed one foot over the other while shuffling or failed to touch the base of the cones. Times were recorded using light gates (Microgate—Witty, Italy), placed at the start/end position. Two trials were completed with 2 min of passive recovery, and better result was taken for analysis [33]. Good test-retest reliability (α = 0.875) was found for this parameter. 

### 2.4. Training Interventions 

Two sessions were conducted to familiarize participants with the training method in order to optimize training adaptations. Two experimental groups (FST and TST) attended 8 weeks of individually supervised strength training, 1–2 training sessions per week, with 12 training sessions in total. The number of training sessions and sets increased progressively throughout the program (Table 1), with at least 48 h rest between sessions. The experimental groups (FST and TST) had the same number of training sessions, sets and repetitions per set during the experimental treatment for each training session (equivolumed training protocols). Moderate inertial load (0.075 kg m^2^) was chosen for half squat and Romanian deadlift for FST group based on findings by Sabido et al. [34] reporting that these loads maximized eccentric overload. All other exercises except Rotational pallof press for both FST and TST participants were conducted with 85% of 1 RM. 

Each training session consisted of 5 drills, with the only difference in the two exercises: while the FST group practiced Romanian deadlift (RDL) and half squats (HS) on the isoinertial device, the TST group practiced half squats (HS) and Romanian deadlift (RDL) with free weights. Two minutes of passive recovery was allowed between exercises and sets. For flywheel exercises each set begins with two submaximal attempts that are not counted in the total number of repetitions, and then the subject continues to exercise with maximum voluntary attempts the required number of repetitions. For half squat exercise, the subject begins with concentric phase caried out from about 90-degree knee angle to near full extension and then continues, without stopping, the phase of eccentric contraction. Participants were briefed to perform the concentric phase with maximum effort, while applying maximal force after the first third of the lengthening phase in order to stop the flywheel at about 90 of knee flexion, thus achieving eccentric overload [21]. It has been recognized that special eccentric strategies are required to apply breaking force over the entire range of motion at certain joint angles to achieve the desired eccentric overload [35]. Romanian deadlift was standing upright holding the Kbar in front and with shoulders width apart. 

For Romanian deadlift, the participants stands on an isoinertial device, placing a Kbar in front of the body, connected to the device by a strap. In the initial position the participant is bent at the hips, the back is straight, the arms are outstretched and the bar is below the knee (knee almost fully extended). The exercise begins by raising the body with maximal voluntary contraction (concentric phase) to an upright position when the strap is stretched to the maximum. It is immediately continued by winding the tape and the participants enters the braking phase in order to stop in the initial position (eccentric phase), after which the next repetition follows without a pause. The bar moves close to the body during exercise.

### 2.5. Statistical Analysis

Data are presented as mean ± standard deviation (SD). Normality of distribution was examined using the Shapiro–Wilk test. Levene’s test for the assessment of homoscedasticity was applied. At pre-test, between-group comparisons were analyzed by univariate analysis of variance (ANOVA) with the factor group (FST, TST and CON), and between-group comparisons under the influence of experimental treatment were analyzed by a two-way ANOVA (3 × 2). Statistical significance was set a priori at *p* ≤ 0.05. Post-hoc test (Least Significant Difference test—LSD) following ANOVA was used to determine the significance of factors interaction. Cohen’s *d* as the measure of the effect size of the mean difference was calculated by subtracting the means and dividing the result by the pooled standard deviation. A Cohen’s d of ≤0.20 = trivial, 0.20–0.60 = small, 0.61–1.20 = moderate, 1.21–2.0 = large and ≥2.01 = very large, as suggested by Hopkins et al. [36]. Data were processed using the SPSS statistical software package, version 20 (Chicago, IL, USA).

## 3. Results

No significant between-group differences were detected in pretest for any variable analyzed. In addition, no meaningful group-by-time interaction was found for sprint 20 m (F = 2.52; *p* = 0.088) (Table 2).

Significant group-by-time interaction was found for ISOMET (F = 6.40, *p* = 0.000), while post hoc analysis revealed differences between FST and CON groups (*p* = 0.014). Comparing the results of the initial and final measurements, FST group had an improvement of 18.7%, (large effect size) the TST group achieved an improvement of 16.6% (large effect size), while the CON groups result was improved by 2.9% (small effect size). Significant group-by-time interaction was found for CMJ (F = 7.45; *p* = 0.001), with post hoc analysis revealing differences between FST and TST group (*p* = 0.006), but also FST and CON (*p* = 0.000) as well as CST and CON (*p* = 0.006). The experimental groups, FST and TST achieved progress of 11.7% (very large effect size) and 6.8% (large effect size), respectively. The CON group had an improvement of 0.3% (trivial effect size). The group-by-time interaction for the 5 m sprint variable (SPR5m) showed a significant difference between groups (F = 7.45; *p* = 0.010). Post hoc analysis showed that there were significant differences between the FST and TST groups (*p* = 0.001) and between FST and CON groups (*p* = 0.000), while there was no significant difference between the TST and CON (*p* = 0.333). Considering the percentage of improvements, 10.3% (very large effect size), 5.9% (moderate effect size) and 3.4% (moderate effect size) were reported for the FST, TST and CON groups, respectively. For the *t*-test, an analysis of the group-by-time interaction showed statistically significant differences (F = 10.46; *p* = 0.000) between groups. Post hoc analysis showed a significant difference (*p* = 0.000) between the FST and CON groups as well as between TST and CON groups (*p* = 0.018). Furthermore, a statistically significant difference was also found between the FST and TST groups (*p* = 0.045). When expressed as a percentage, the reported improvements were 2.4% (very large effect size) for the FST group, 1.4% (large effect size) for the TST group and 0.6% for the CON group (moderate effect size).

## 4. Discussion

It has been proposed that flywheel training is an efficient method for enhancing a myriad of fitness attributes in team sport athletes [13]. However, studies exploring the effectiveness of flywheel training with basketball athletes is lacking. Therefore, the aim of the present investigation was to compare the in-season effects of equivolumed flywheel vs. traditional strength training on lower body strength, countermovement jump, change of directions ability and sprint performance in well-trained young basketball players. The results of this research indicate that there were no differences in strength improvements for two experimental protocols while flywheel training was proved to be superior for developing agility, vertical jump and 5 m sprint time. Flywheel group displayed significantly higher improvements in strength, vertical jump, 5 m sprint time and change of direction ability compared to control group. Players from traditional strength training group showed better results in vertical jump and change of direction ability compared to players from control group. Interestingly, adding one/two sessions a week of flywheel training appears to be an appropriate strategy for enhancing lower body strength during competitive period in young basketball players while adding equivolumed traditional strength training seems less effective. Finally, neither training modality was proved effective for enhancing 20 m sprint performance. 

Although this type of practice is very popular in the last decades [13], scanty studies have compared the effects of flywheel and traditional weight training on performance in athletic population [17,19,37], and generally presented data similar to our study findings. In a six week study by Maroto-izguierdo et al. [19], 15 flywheel training sessions (4 × 7 maximal intensity half squats done with 0.145 kg·m^2^ moment inertia) produced superior improvements (*p* < 0.05–0.001) compared to traditional weight training (4 × 7 leg presses with load corresponding to 7 repetitions maximum (7 RM) for each set) for vertical jump (9.8% vs. 3.4%), change of direction ability (−7% vs. −4.4%) but also 20 m sprint time (−10% vs. −5.1%) in professional handball players. In addition, no significant differences between strength training modalities were observed for maximum strength improvement (12.2% and 7.9% for flywheel and traditional weight training, respectively). The outcomes of the 8 week Corratela et al. [17] study demonstrated that flywheel strength training performed once per week with up to 6 sets of 8 repetitions of squats produced superior improvements to equivolumed traditional weight training (80% of 1 RM) for change of direction ability (−7% vs. −2%, respectively) and 20 + 20 m sprints (−4% vs. −1%, respectively) but not for jumping (squat jump and countermovement jump) and sprinting abilities (10 m sprint and 30 m sprint) in professional soccer players. Furthermore, lower body strength increased significantly and similarly in both groups. Finally, effects of flywheel and traditional strength training on 10-m sprint, CMJ and lower body strength (1 RM squat) were examined on 38 active male football players by Sagelv et al. [37]. During six weeks of intervention (2 sessions per week), both flywheel and traditional strength training progressively increased squat exercise from 3 sets with 6 repetitions (week one) to 4 sets with 4 repetitions (week six). Flywheel group performed exercise with individually adjusted inertia enabling high power outputs (>4 watts·kg^−1^) while traditional strength training comprised of 4 sets with 4 repetitions (85% of 1 RM) was performed with maximum intended velocity. In addition, an equivolumed Nordic hamstring exercise was included for both groups with three sets of 4–10 repetitions to counteract expected strength gains in quadriceps muscle. Both groups significantly improved CMJ (9% and 8% for flywheel and traditional strength group, respectively) and identically decreased 10 m sprint time (2%) without between group differences for either variable. Interestingly, traditional strength training was proved superior to flywheel training in improving lower body strength (46% vs. 19%, respectively), with the noteworthy observation that traditional weight training was conducted with high loads (85%) and maximal intended velocity which is likely the primary reason for observed improvements [38]. Collectively, the aforementioned study corroborates our study findings that flywheel training induces superior power-related performance but not strength outcomes to traditional weight training modalities in the athletic population. 

In addition, these studies suggest that flywheel training is potent tool for strength and power related performance attribute improvements in the well-trained population, which is broadly supported with several other studies. Indeed, a recent meta-analysis reported flywheel-training induced strength improvements, but also no difference in strength increase after flywheel vs. traditional weight training [39]. Askling et al. [16] and deHoyo et al. [14] after 10 weeks of flywheel training (16 and 17 sessions, respectively) in elite soccer players (seniors and juniors, respectively) reported significant strength (*p* ≤ 0.05; 19% and 15% for eccentric and concentric strength, respectively) and 30 m sprint time (*p* ≤ 0.05; 2.4%) improvements as well as vertical jump (7.6%) and sprint time (20 m sprint, 1.5%; and 10 m flying sprint, 3.3%) improvements, respectively. In addition, six weeks of flywheel training, performed twice a week, has been shown to induce statistically higher improvements in squat jump and drop jump performance as well as change of direction ability compared to volume-matched plyometric training in well-trained junior soccer players [18]. On the contrary, implementing one flywheel training session per week for 7 weeks was found ineffective for lower body strength (1 RM in the half squat), 20 m sprint time, and CMJ improvements in professional handball players [20], suggesting that more than one flywheel training per week, with up to 4 sets (7 reps), is needed for substantial power-related performance improvements in the athletic population [1]. Indeed, Corattela et al. [17] reported significant improvements in change of direction ability and vertical jump performance (SJ and CMJ) after 6 weeks of flywheel strength training performed just once per week but with higher number of sets and reps (6 and 8, respectively). Collectively, these data support efficacy of flywheel training for improving broad range of strength and power -related performance attributes in well trained population, with noteworthy caution considering threshold load that needs to be met in order to obtain significant improvements. Clearly, additional investigations about the topic are warranted.

It is interesting to note that we found no significant effects of flywheel nor traditional strength training on 20 m sprint performance in our participants. Somewhat in line with our findings, no change in 20 m sprint time was reported after horizontal flywheel training in physically active men [40]. It has been previously reported that low-velocity strength training may not be effective in improving sprinting ability in adolescents, especially well-trained athletes [41]. However, two-to-three flywheel sessions per week has been proven to increase the sprinting ability in handball players [19]. In addition, sprint time (10 and 20 m) significantly improved following 9 weeks of strength training in youth soccer players [42]. We can speculate that our study results are on one side consequence of the training status of our participant (well trained), as it has been shown that trained adolescents displayed hindered improvements in sprint outcomes with strength training compared to untrained one [43]. On the other hand, training and testing specificity could be also responsible as upward force-vector application during training likely play an important determinant in inducing specific functional adaptations [27]. In addition, 20 m sprint is rarely seen in a basketball game and practice and consequently sprint tests over shorter distances (5–10 m) might be more specific with acceleration and deceleration, rather than speed, as a far stronger predictor of basketball performance [44,45].

Although beyond the scope of this study, mechanisms that enables reported improvements in strength and power related performance outcomes should be concisely hypothesized. Flywheel training enables maximal force output throughout the entire concentric part of movement, but also short periods of overload in the eccentric phase of movement [13]. As exercise intensity has been acknowledged as a major determinant for strength training induced adaptations [46,47]. It can be speculated that this flywheel specific loading pattern (concentrically maximally loaded-eccentrically overloaded) is most likely responsible for superior effects for power-related performance outcomes in our study. Furthermore, eccentric overload induced specific neuromuscular adaptations such as dampened motor recruitment [48] with preferential recruitment of high threshold motor unit and higher cortical activity [49]. Finally, it has been reported that increase in eccentric phase force output leads to increase in following concentric phase force output [50,51,52]. Collectively, this physiological distinctiveness supports our study findings and the beneficial use of flywheel training to optimize strength and power adaptations in young basketball athletes. 

Several limitations of the study should be highlighted. We did not monitor load of regular basketball practice done by all participants with their respective coaches, which could somewhat blur the picture of obtained strength training effects. In addition, this study engaged male trained basketball players, without preceding experience in the flywheel training. Accordingly, the results may not translate to flywheel-experienced athletes. Finally, our study lasted for 8 weeks only, while comparative investigations with traditional strength modalities of longer durations are needed.

## 5. Conclusions

In summary, eight weeks of flywheel training with 1–2 sessions per week, including up to 4 sets of 8 repetitions of the half squat and Romanian deadlift exercises performed with maximum concentric intensity produces superior enhancement in vertical jump, 5 m sprint time and change of direction ability to equivolumed traditional strength training in well-trained young basketball players. In addition, both strength training modalities were equally effective in maximal strength gains. Therefore, low-volume/high-intensity flywheel strength training seems to be an efficient tool to induce strength and power-related adaptations in well-trained young basketball players.

## Figures and Tables

**Table 1:** Training program for flywheel (FST) and traditional strength training (TST) groups.

FST	TST
Week 1–2	Week 1–2
Number of training sessions: 1	Number of training sessions: 1
One-arm dumbbell row (2 × 8)	One-arm dumbbell row (2 × 8)
Rotational pallof press 2 × (2 × 12–15)	Rotational pallof press 2 × (2 × 12–15)
Biceps curls + upright row complex (2 × 8)	Biceps curls + upright row complex (2 × 8)
Half squat on isoinertial device (2 × 8)	Half squat with free weights (2 × 8)
Romanian Deadlift (RDL) on isoinertial device (2 × 8)	Romanian deadlift (RDL) with free weights (2 × 8)
Week 3–4	Week 3–4
Number of training sessions: 1	Number of training sessions: 1
One-arm dumbbell row (3 × 8)	One-arm dumbbell row (3 × 8)
Rotational pallof press 2× (3 × 12–15)	Rotational pallof press 2 × (3 × 12–15)
Biceps curls + upright row complex (3 × 8)	Biceps curls + upright row complex (3 × 8)
Half squat on isoinertial device (3 × 8)	Half squat with free weights (3 × 8)
Romanian Deadlift (RDL) on isoinertial device (3 × 8)	Romanian deadlift (RDL) with free weights (3 × 8)
Week 5–6	Week 5–6
Number of training sessions: 2	Number of training sessions: 2
One-arm dumbbell row (3 × 8)	One-arm dumbbell row (3 × 8)
Rotational pallof press 2 × (3 × 12–15)	Rotational pallof press 2 × (3 × 12–15)
Biceps curls + upright row complex (3 × 8)	Biceps curls + upright row complex (3 × 8)
Half squat on isoinertial device (3 × 8)	Half squat with free weights (3 × 8)
Romanian Deadlift (RDL) on isoinertial device (3 × 8)	Romanian deadlift (RDL) with free weights (3 × 8)
Week 7–8	Week 7–8
Number of training sessions: 2	Number of training sessions: 2
One-arm dumbbell row (4 × 8)	One-arm dumbbell row (4 × 8)
Rotational pallof press 2 × (4 × 12–15)	Rotational pallof press 2 × (4 × 12–15)
Biceps curls + upright row complex (4 × 8)	Biceps curls + upright row complex (4 × 8)
Half squat on isoinertial device (4 × 8)	Half squat with free weights (4 × 8)
Romanian Deadlift (RDL) on isoinertial device (4 × 8)	Romanian deadlift (RDL) with free weights (4 × 8)

**Table 2:** Between-group differences in selected variables with % of improvement and Cohen’s effect size (d).

	FST		TST						CON				
	IN	FIN	%	d	IN	FIN	%	d	IN	FIN	%	d	*p*
ISOMET	92.33 ± 10.57	109.83 ± 7.81	18.7	1.883	90.25 ± 10.35	105.25 ± 9.36	16.6	1.520	92.42 ± 4.08	94.33 ± 3.28	2.9	0.516	0.000 ^‡^
CMJ	52.36 ± 3.33	59.29 ± 2.97	11.7	2.196	51.45 ± 3.61	55.22 ± 3.07	6.8	1.125	50.77 ± 2.53	50.92 ± 2.56	0.3	0.059	0.001 ^†,‡,∆^
SPR5m	1.16 ± 0.04	1.04 ± 0.02	10.3	3.795	1.18 ± 0.07	1.11 ± 0.05	5.9	1.151	1.18 ± 0.03	1.14 ± 0.06	3.4	0.843	0.010 ^†,‡^
SPR20m	3.20 ± 0.11	3.07 ± 0.09	4.1	1.294	3.24 ± 0.10	3.13 ± 0.11	3.4	1.046	3.21 ± 0.051	3.19 ± 0.56	0.6	0.05	0.088
*t*-test	10.07 ± 0.10	9.83 ± 0.07	2.4	2.781	10.04 ± 0.09	9.90 ± 0.08	1.4	1.644	10.12 ± 0.07	10.06 ± 0.06	0.6	0.92	0.000 ^†,‡,∆^

ISOMET—isometric strength test; CMJ—countermovement jump test; SPR5m—20 m sprint test; SPR20m—5 m sprint test; *t* test—agility *t*-test; IN—initial tests result ± standard deviation; FIN—final test result ± standard deviation; %—percentage of improvement; *p*—level of statistical significance; ^†^—statistically significant difference between FST and TST group; ^‡^—statistically significant difference between FST and CON group; ^∆^—statistically significant difference between TST and CON group.
